# Primary malignant lymphoma combined with clinically “silent” pheochromocytoma in the same adrenal gland

**DOI:** 10.1186/s12957-015-0711-6

**Published:** 2015-09-30

**Authors:** Anna Babinska, Rafał Peksa, Krzysztof Sworczak

**Affiliations:** Department of Endocrinology and Internal Medicine, Medical University of Gdansk, ul. Dębinki 7, 80-288 Gdańsk, Poland; Department of Pathology, Medical University of Gdansk, ul. Dębinki 7, 80-288 Gdańsk, Poland

**Keywords:** Adrenal lymphoma, Pheochromocytoma, Treatment

## Abstract

An increased number of adrenal tumors are now diagnosed due to the increased number of abdominal CT scans being performed. We present the first case of malignant lymphoma combined with clinically “silent” pheochromocytoma in the same adrenal gland. An abdominal CT scan demonstrates unilateral adrenal lesion which suggests pheochromocytoma or adrenal carcinoma. Laboratory examinations revealed a slight increase of 24-h urine vanillylmandelic acid and 24-h urinary methanephrine excretion. Histological examination revealed two intermingled tumor cell proliferations—diffuse B cell lymphoma and pheochromocytoma.

Unexpected coexistence of catecholamine-producing tumor with the other adrenal lesion can lead to serious complications of diagnosis and treatment. The adequate preparation for surgery can protect patient from threatening catecholamine crisis.

## Background

Lymphoma may occasionally involve the adrenal glands, but primary adrenal lymphoma (PAL) is very rare [1]. Autopsy studies have revealed that 25 % of patients with non-Hodgkin’s lymphoma have involvement of the adrenal gland during their disease. However, lymphomas originating from the endocrine glands account for only 3 % of extra nodal lymphomas and usually appear only in the thyroid gland [2, 3].

Combined adrenal neoplasms are also rare entities. We report herein, to the best of our knowledge, the first case of collision tumor composed of a primary adrenal lymphoma and a clinically “silent” pheochromocytoma.

## Case presentation

A 57-year-old man was admitted to the emergency department of Medical University of Gdansk with right abdominal pain suggesting cholelithiasis. There was a history of nausea, fatigue, and weight loss of 10 kg in the last 3 months. The patient had no other medical history.

Primary physical examination revealed a normal blood pressure of 130/70 mmHg, pulse of 90/min, and no fever. The abdominal examination revealed a painful mass in the right side without hepatosplenomegaly. Neither signs of hypercortisolism nor lymphadenopathy were found.

A contrast enhanced computer tomography (CT) was performed and showed a 13.2 × 11 cm solid right adrenal mass. The mass was homogenous with high Housfield score (51 HU) in basic condition, and contrast washout rate was 30 % after 10 min. No other abdominal lesions or lymphadenopathy were detected. Initial laboratory findings revealed a normal complete blood count, but erythrocyte sedimentation rate (ESR) was 74 mm/h (N, 0–20 mm/h), C-reactive protein (CRP) was 48 mg/L (N, 0–6 mg/L), and lactate dehydrogenase (LDH) was 240 IU/L (N, 105–240 IU/L). Hormonal laboratory tests showed a 24-h urine vanillylmandelic acid (VMA) excretion of 10.2 mg/24 h (N, 4–8 mg/24 h) and a 24-h urinary methanephrine excretion of 395 μg/24 h (N, <350 μg/24 h). Serum androstendione was slightly elevated to 4.58 ng/mL (N, 0.3–3.5 ng/mL) (see Table 1).

**Table 1:** Laboratory results of presenting patient

Disease	Androstendione	DHEA-S	Cortisol urinary excretion	Cortisol 8.00 a.m./8.00 p.m.	ACTH 8.00/20.00	VMA/metanephrine daily urinary excretion	ESR	CRP	LDH
Basal	4.58	33.7	140	332/133	13/<10	10.2/395	74	48	240
Progression	–	–	–	149/–	102/–	–/254	98	57	–

Normal ranges: serum LDH, 105–240 IU/L; serum androstendione, 0.3–3.5 ng/mL; serum DHEA-S, 34–430 μg/dL; 24-h cortisol urinary excretion, 12–486 nmol/24h; serum cortisol: 8.00 a.m. 101–536 nmol/dL; plasma ACTH, 15–46 pg/mL; 24-h metanephrines excretion, <350 μg/24h; 24-h vanillylmandelic acid (VMA) excretion, 4–8 mg/24h; serum erythrocyte sedimentation rate (ERS), 0–20 mm/h; serum C-reactive protein (CRP), 0–6 mg/L

The results of hormonal and image findings firstly suggested malignant adrenal lesion or pheochromocytoma (PHEO) and were not suggestive of lymphoma. A slight increase of catecholamine level with the absence of clinical symptoms of PHEO inclined us to small doses of alpha-blockers only—doxazosin 4 mg/24 h per os to control paroxysmal blood pressure rise during the surgery. Consequently, the patient underwent open right adrenalectomy. The mass was found to press the neighboring organs and vessels without evident infiltration. The tumor resection with right nephrectomy was performed.

### Pathological findings

Macroscopically gray creamy tumor measuring up to 7 cm in greatest dimension was found in the right adrenal gland infiltrating periadrenal adipose tissue and the cortical part of the kidney. Renal parenchyma infiltration measured up to 3.5 cm.

**Fig. 1:**
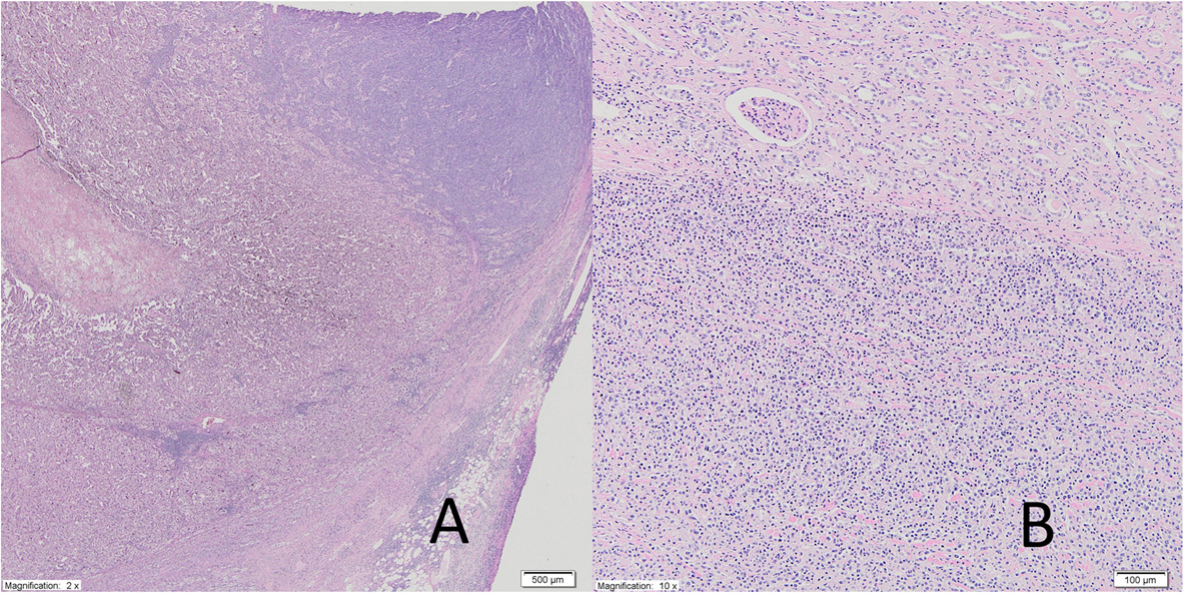
Pheochromocytoma is present on the left with a diffuse area of the right. The diffuse area is comprised predominantly of large lymphoid cells, so a separate diagnosis of DLBCL is made (**a**, magnification ×2). Adrenal tumor infiltrates periadrenal adipose tissue and the cortical part of kidney (**b**, magnification ×10)

Gross lesion found in a section of the gland consisted of two parts: gray creamy tumor which destroyed the adrenal gland and the yellowish-brown spherical focus 1.8 cm in diameter which adheres to the adrenal mass. Adrenal tumor infiltrates periadrenal adipose tissue and the cortical part of the kidney (Fig. 1).

**Fig. 2:**
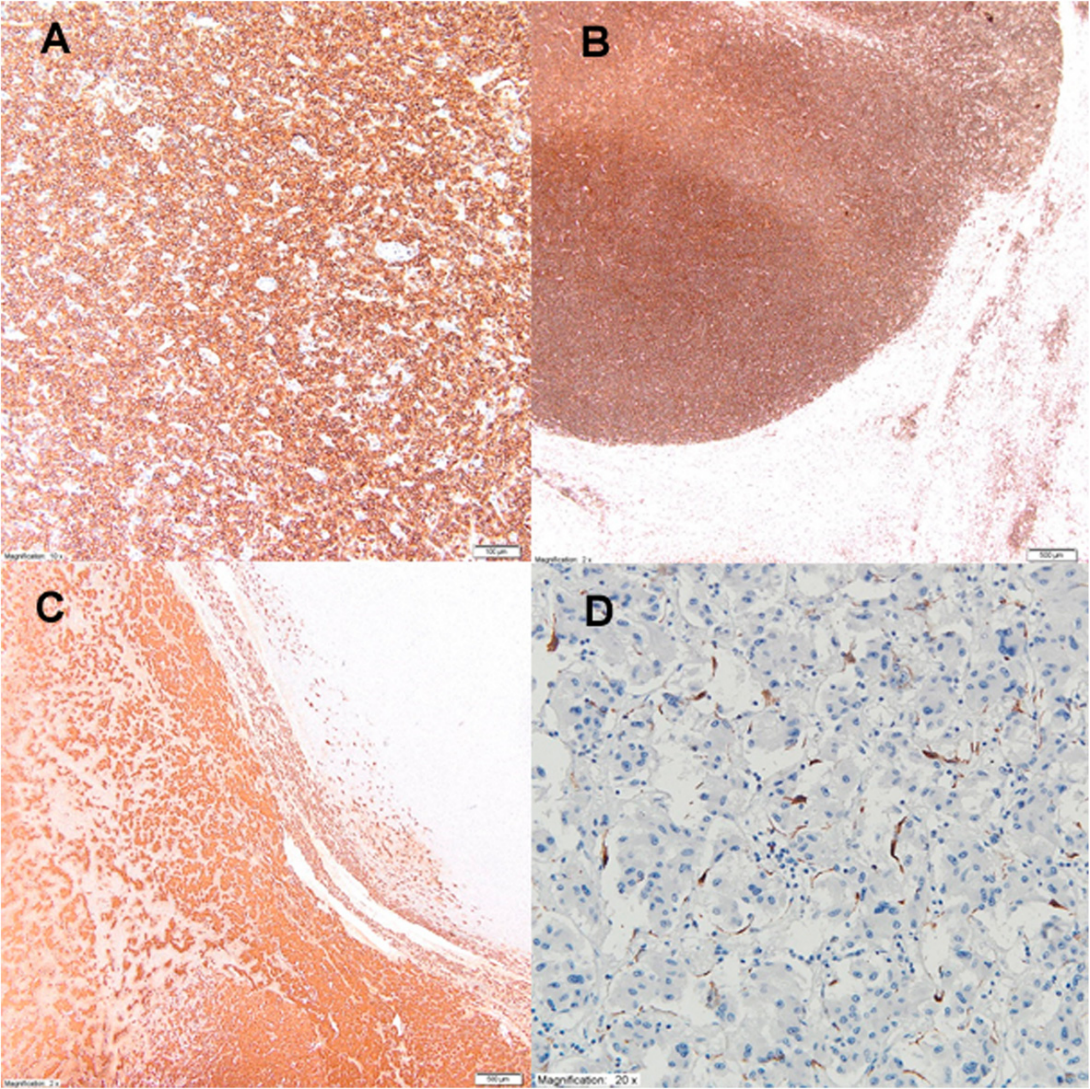
In the immunohistological study, tumor cells were positive for leukocyte antigen CD20(+) (**b**, magnification ×10) and CD45(+) (**c**, magnification ×2), and focus of pheochromocytoma was positive for chromogranin A(+) (**a**, magnification ×2) and S-100 (**d**, magnification ×20)

Microscopically, samples from the adrenal tumor revealed diffuse large B cell lymphoma (DLBCL) with the following immunophenotype: CD20(+), CD45(+), CD3(−), Ki67-50 %, CD5(−), CD10(−), BCL6(−), MUM1(−), and cyclin D1(−) (Fig. 2). Lymphoma infiltration exceeded the adrenal capsule and infiltrated adipose tissue and renal parenchyma. The microscopic picture of brown focus was consistent with the diagnosis of pheochromocytoma (chromogranin A(+) and S-100(+); Fig. 2).

Conclusion: microscopic examination revealed DLBCL and 1.8 cm focus of PHEO.

### Post-operative course

Before the therapy began, whole body CT scans and bone marrow aspiration biopsy was obtained. The absence of any lymphadenopathy and no bone marrow involvement ruled out a secondary lymphoma. The patient received chemotherapy: cyclophosphamide (750 mg/m^2^), doxorubicin (50 mg/m^2^), vincristine (1.4 mg iv), and prednisolone (100 mg po).

After a period of 3 months, a control CT scan showed progression of the disease with further enlargement of the left adrenal mass 11 × 10 × 15 cm. The mass was homogenous and HU score was high. The 24-h urinary excretion of metabolites of catecholamines was normalized to 254 μg/24 h after surgery and remained normal during the progression of the disease (see Table 1).

Laboratory examination showed hyponatremia—sodium level of 129 mmol/L (N, 137–145 mmol/L) and potassium level of 4.93 mmol/L (N, 3.6–5.0 mmol/L). Due to the symptoms of hypotension, hyponatremia, and neoplasm enlargement of the left adrenal gland, adrenal insufficiency was suspected. The baseline serum cortisol was 149 nmol/L (N, 101–536 nmol/L), and serum ACTH was elevated to 102 pg/mL (N, 15–46 pg/mL), which indicated a primary adrenal insufficiency (see Table 1). The patient was treated with intravenous hydrocortisone (100 mg), and after the complete remission of symptoms and normalization of electrolytes, oral hydrocortisone substitution (30 mg/day) and fludrocortisone (0.05 mg/day) were continued. Bone marrow aspiration biopsy showed atypical lymphocyte infiltration. Chemotherapy was continued but the patient died of sepsis after 10 months.

## Discussion

Lymphoma may spread to any part of the body, and involvement of the adrenals in the course of malignant lymphomas is reported in 25 % of autopsies [1]. PAL is a rare neoplastic disease, and only 187 cases have been reported in the literature so far [1–5].

Autoimmune adrenalitis has been proposed as an etiology of PAL [3]. Immune dysfunction such as history of cancer (15 %), human immunodeficiency virus (HIV, 4 %), Epstein Barr virus infection, or autoimmune disorder (13 %) may be a predisposing factor in same patients [3].

Combined adrenal neoplasms are very rare [4, 5]. Most published cases describe pheochromocytoma coexisting with ganglioneuroma or ganglioneuroblastoma [2, 5]. Occasionally, PHEO with malignant peripheral nerve sheath tumor was identified [4, 5]. A collision tumor comprising a primary adrenal lymphoma and subclinical PHEO has never been reported before.

PAL usually manifests itself with bilateral, large masses, sometimes accompanied by adrenal insufficiency [2, 5]. Among clinical symptoms are fever, weight loss, and local pain [1, 2, 5]. In the described case, PAL was diagnosed with abdominal pain and no clinical symptoms of “silent” pheochromocytoma.

Although imaging modalities help in the diagnosis of the adrenal mass, making a firm diagnosis is difficult particularly in unilateral involvement. Fine needle aspiration biopsy or adrenalectomy is performed when histopathological diagnosis is required [6].

Over 12 % of patients with incidentally found pheochromocytoma may be asymptomatic and normotensive, so all patients with adrenal incidentaloma should undergo biochemical testing for pheochromocytoma [6]. Normal results of 24-h urinary metanephrine excretion rule out PHEO [6].

Unexpected coexistence of PHEO with the other adrenal lesion can lead to serious complications of potential biopsy, in cases of suspicion of metastases or PML [6, 7]. These procedures provided in patient with pheochromocytoma may cause life threatening catecholamine crisis and should be performed after exclusion of PHEO [7, 8].

Many drags can cause adverse reaction in patients with PHEO. Glucocorticoids can induce catecholamine synthesis in PHEO cell culture, inducing catecholamine biosynthetic enzymes and increase the release of catecholamines from perfused canine adrenal glands [7, 8]. Glucocorticoids should be avoided or administered with vigilance in patients suspected of PHEO [8].

In spite of absence of clinical symptoms of PHEO, compared to a slight increase of the 24-h urinary catecholamine excretion, we withdraw from the adrenal biopsy and dexamethasone suppression test.

Most authors recommend surgery for hormonal active adrenal tumors or tumors larger than 4 cm [9, 10]. In this case, several preoperative findings were suggestive of primary malignant adrenal neoplasm: production of adrenal androgens (androstendione) and tumor size >4 cm with delayed contrast washout in CT scan (less than 50 % after 10 min).

Hormonal suspicion of PHEO inclined us to pharmacological treatment before surgery.

Any patient with suspected PHEO should undergo preoperative alfa—adrenergic receptor blockade [11]. Adequate preparation for surgery aims at lowering blood pressure, slowing down the heart rate, and controlling paroxysmal blood pressure rise and other features of catecholamine overload [11]. Consequently, we qualified our patient for open adrenalectomy. Surgery is mainly used to obtain the pathological tissue for diagnosis and does not affect the prognosis in PML [1, 3, 11].

The two most common WHO 2008-defined PAL subtypes are DLBCL—78 % and peripheral T cell lymphoma—7 % [1]. Histopathological examination of our patient revealed DLBCL with the unexpected focus of clinically “silent” pheochromocytoma.

Most patients with PAL have bilateral disease on admission, and most of them have adrenal insufficiency subsequent to destruction of more than 90 % of adrenal parenchyma by the disease [5, 12, 13]. Because of unilateral adrenal involvement, the adrenal function was normal in the early phase. Due to disease progression, here, symptoms of adrenal insufficiency appeared after 3 months. The proper blood pressure and lack of other symptoms of adrenal insufficiency in the initial period of the disease could be associated with presence of normal adrenal parenchyma in the other gland.

Most patients with PAL have a limited time of survival. It has been reported in the literature that advanced age, primary adrenal insufficiency, tumor size, LDH, and serum soluble interleukin-2 receptor level (sIL-2R) and involvement of other organs are poor prognostic indicators [1, 3, 5]. Unilateral adrenal lymphoma may have a longer survival rate [3]. Complete remissions have been reported only in few cases [3, 14, 15].

## Conclusions

PAL should be kept in mind in the differential diagnosis of patients with unilateral adrenal masses without nodal involvement in the absence of other malignancies [1, 13, 14]. Unexpected coexistence of catecholamine-producing tumor complicates diagnosis based on adrenal biopsy [4, 15]. The adequate preparation for surgery can protect patient from threatening catecholamine crisis [11, 15].

To the best of our knowledge, PAL and PHEO coexistence in one adrenal gland has never been described.

## Consent

Written informed consent was obtained from the patients for publication of this report and any accompanying images.
